# Evolution of Body Elongation in Gymnophthalmid Lizards: Relationships with Climate

**DOI:** 10.1371/journal.pone.0049772

**Published:** 2012-11-14

**Authors:** Mariana B. Grizante, Renata Brandt, Tiana Kohlsdorf

**Affiliations:** Department of Biology, Faculdade de Filosofia Ciências e Letras de Ribeirão Preto, Universidade de São Paulo, Ribeirão Preto, São Paulo, Brazil; Fred Hutchinson Cancer Research Center, United States of America

## Abstract

The evolution of elongated body shapes in vertebrates has intrigued biologists for decades and is particularly recurrent among squamates. Several aspects might explain how the environment influences the evolution of body elongation, but climate needs to be incorporated in this scenario to evaluate how it contributes to morphological evolution. Climatic parameters include temperature and precipitation, two variables that likely influence environmental characteristics, including soil texture and substrate coverage, which may define the selective pressures acting during the evolution of morphology. Due to development of geographic information system (GIS) techniques, these variables can now be included in evolutionary biology studies and were used in the present study to test for associations between variation in body shape and climate in the tropical lizard family Gymnophthalmidae. We first investigated how the morphological traits that define body shape are correlated in these lizards and then tested for associations between a descriptor of body elongation and climate. Our analyses revealed that the evolution of body elongation in Gymnophthalmidae involved concomitant changes in different morphological traits: trunk elongation was coupled with limb shortening and a reduction in body diameter, and the gradual variation along this axis was illustrated by less-elongated morphologies exhibiting shorter trunks and longer limbs. The variation identified in Gymnophthalmidae body shape was associated with climate, with the species from more arid environments usually being more elongated. Aridity is associated with high temperatures and low precipitation, which affect additional environmental features, including the habitat structure. This feature may influence the evolution of body shape because contrasting environments likely impose distinct demands for organismal performance in several activities, such as locomotion and thermoregulation. The present study establishes a connection between morphology and a broader natural component, climate, and introduces new questions about the spatial distribution of morphological variation among squamates.

## Introduction

The general shape of a given body is recognized by its distribution along a three-dimensional Cartesian space. The morphological changes that equally increase an object in these three axes will result in a larger body with the same original shape; in contrast, when variation occurs mostly in one of these three dimensions (e.g., along the horizontal axis) the result may be a very distinct shape, such as an elongated body. Elongated animal body shapes evolve through increases in length (given, for example, by the addition of vertebrae along the trunk [1]–[3]), which can be either paralleled by changes in the trunk diameter [2] or coupled with decreases in the trunk height or width [4], [5]. Changes leading to the evolution of elongated forms have been identified in basically all vertebrate lineages (e.g., fishes [4]–[6], amphibians [7], [8], squamates [3], [9]–[12] and mammals [13], [14]) and often also involve a reduction or loss of the locomotor appendages [2], [6], [9]–[12], [15].

The recurrent evolution of elongated body shapes in vertebrates has intrigued biologists for decades [2], [7]–[16]. Squamata, in particular, has been used as a model system for detecting general patterns toward the evolution of serpentiform morphologies [9], [16], which are characterized by elongated trunks and reduced or absent limbs [3], [9], [10]. There are several aspects that may explain how the evolution of body elongation relates to environmental traits. Studies on functional morphology suggest that long and thin, limbless bodies enhance the burrowing performance during subterranean locomotion such that the evolution of serpentiform squamates would be favored in fossorial lineages [2], [17]. Even so, not all elongated squamates are fossorial, and there are possibly other parameters that trigger body shape diversification. For example, ecological interactions (e.g., competition and invasion of available niches) have been recently claimed to be relevant factors for the origin of elongated squamates because such interactions might explain the existence of two elongated ecomorphs in the lineage: the short-tailed fossorial species and the long-tailed surface-dwelling forms [9], [10].

Regardless of the selective pressures that may be related to the evolution of elongated forms in Squamata, these conspicuous changes in body shape likely affect the interactions between the organism and its surrounding environment. The association between morphology and ecology has been identified in several squamate lineages [18]–[23]. Nevertheless, our current concept of the environment can be extended to encompass climate and thus improve our knowledge of the environmental effects on morphological variation. Thanks to recent geographic information system (GIS) techniques, the climate parameters obtained from specimen localities are now easily incorporated into studies in evolutionary biology [24]. Indeed, this approach has been reported in recent articles, suggesting that the general patterns of variation in some phenotypic traits of squamates are associated with climate [22], [25]–[27]. However, it is important to emphasize that none of these studies have focused on the evolution of elongated body forms. The relationships between climate and the evolution of body shape may be predicted by the direct and indirect effects that climatic components likely have on biological traits [28]. For example, in vertebrate ectotherms, temperature and precipitation are directly related to thermoregulation patterns and rates of water loss, which may be strongly dependent on body size and form [29]. Moreover, temperature and precipitation likely determine other environmental characteristics, such as soil texture, substrate coverage and plant primary productivity (and consequent prey availability) in a given habitat [24], [26], [30]. Together, these environmental factors define some of the selective pressures acting during morphological evolution such that new associations between environment and morphology may be revealed when climatic variation is included in this evolutionary equation.

The associations between variation in body shape and climate are the focus of the present study. Specifically, we tested for correlations between the morphological traits that determine body shape and climatic parameters using gymnophthalmid lizards as a model system. The family Gymnophthalmidae is a good system to test for associations between morphological and climatic variations because it is composed of lineages that represent a gradient of body form ranging from lacertiform to serpentiform shapes [31]. These lizards are broadly distributed and occupy diverse habitats and, thus, are exposed to a wide range of climates [3], [31]–[33]. Moreover, there are robust phylogenetic hypotheses available for this group [31], [34], which allows the formal investigation of evolutionary associations. We first tested the hypothesis that body form has evolved to become elongated in Gymnophthalmidae, based on linear morphological traits. This hypothesis was tested using a phylogenetic Principal Component Analysis (PCA) [35], which identified clusters of species based on body shape and elongation. We then tested the hypothesis that the variation in body shape (particularly body elongation) is associated with climatic parameters in gymnophthalmid lizards. This hypothesis was tested using a phylogenetic covariance analysis between the environmental traits and the morphological component that resulted from our first analysis. These complementary approaches are innovative by adding the dimension of climate to the investigation of the evolution of body elongation in Squamata.

## Results

**Figure 1:**
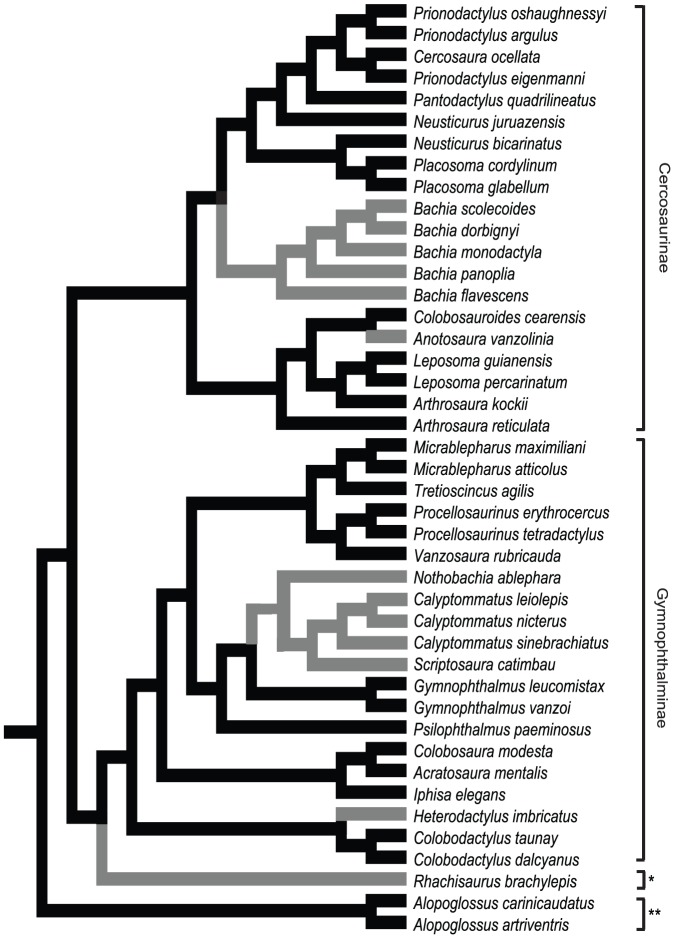
Topology of Gymnophthalmidae used in the phylogenetic analysis. Branch colors represent the elongation groups detected using the morphological component (morphPC): less-elongated species are shown in black and more-elongated species in gray. Asterisks represent the subfamilies Rhachisaurinae (*) and Alopoglossinae (**); taxonomy adopted follows [31].

**Figure 2:**
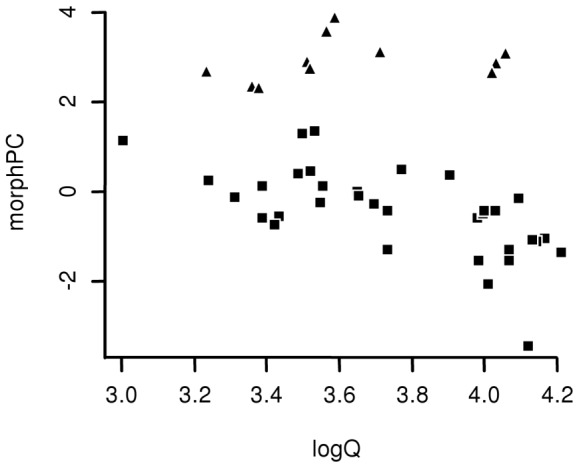
Relationships between aridity index (logQ) and body elongation (given by morphPC) in gymnophthalmid lizards. MorphPC = morphological principal component. Symbols represent elongation groups: triangles correspond to the more-elongated species, and squares indicate less-elongated species.

The present study had two major goals. We first identified a composite variable clustering the linear morphological traits that likely changed during the evolution of elongated morphologies in Gymnophthalmidae. We retained only one morphological component (hereafter referred to as morphPC) on a phylogenetic PCA, which had eigenvalue equal to 6.24 and explained 78% of the morphological variation (Table 1) of the 45 gymnophthalmid species that are listed in Figure 1. MorphPC presented high positive loadings for trunk length and high negative loads for the remaining morphological traits (head length, height and width, pelvic girdle height and width and anterior and posterior limb lengths; Table 1). Thus, in Gymnophthalmidae, increases in trunk length were simultaneously coupled with limb shortening and body narrowing, the latter represented by a decreased width and height of the head and the pelvic girdle. The use of morphPC clustered gymnophthalmid species into two groups based on the degree of body elongation: less and more elongated gymnophthalmids (Figure 2). The species classified as more elongated included lineages characterized by extreme limb reduction, such as *Bachia* (Cercosaurinae, [31]), *Scriptosaura*, *Calyptommatus* and *Nothobachia* (Gymnophthalminae, [31]), together with the pentadactyls *Anotosaura vanzolinia* (Cercosaurinae, [31]) and *Heterodactylus imbricatus* (Gymnophthalminae, [31]) and the tetradactyl *Rhachisaurus brachylepis* (Rhachisaurinae, [31]).

**Table 1:** Variable loadings resulting from a phylogenetic Principal Component Analysis (PCA) performed on the morphometric variables measured in gymnophthalmids.

Morphological variable	morphPC
Trunk length	0.84
Head length	−0.91
Head height	−0.91
Head width	−0.89
Pelvic girdle height	−0.86
Pelvic girdle width	−0.88
Anterior limb length	−0.86
Posterior limb length	−0.91
Eigenvalue/% variation explained	6.24/78%

MorphPC = morphological principal component.

**Figure 3:**
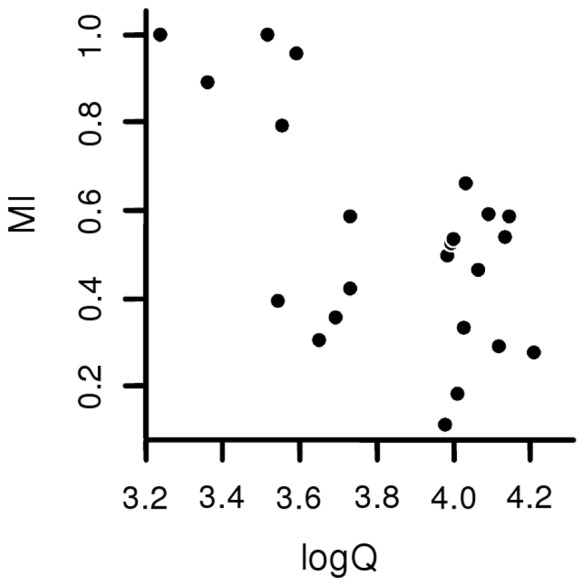
Linear regression between microhabitat index (MI; variable obtained from Barros et al., 2011) and aridity index (logQ) in gymnophthalmid lizards.

The dichotomy that describes gymnophthalmid species as less or more elongated was then used to test for associations between climatic parameters and morphological patterns, which was the second major goal of this study. We are reporting the results for the best fit phylogenetic linear models (lower AICc, Table 2), but results obtained for all the 31 models tested are synthesized in Table S3. Regardless of the morphological categorization, body elongation in Gymnophthalmidae increased with aridity (see Figure 2 and Table 2). Besides body elongation, the use of substrates that offer greater resistance for locomotion, by gymnophthalmid lizards, also increased with aridity (Fig. 3; slope = −0.6037, p<0.001).

**Table 2:** Best linear models testing the effects of aridity index (logQ) and elongation groups (EgroupPC) on the morphological component (morphPC).

Model	Parameter	slope	p	λ
morphPC∼log.Q+EgroupPC	log Q	−1.283	<0. 001*	0.961
	EgroupPC	2.430	<0. 001*	
morphPC∼log.Q×EgroupPC	log Q	−1.452	<0. 001*	0.945
	EgroupPC	−1.300	0.656	
	log.Q×EgroupPC	1.057	0.197	

Significant values (P<0.05) are indicated with an asterisk (*).

## Discussion

The present study investigated how the morphological traits that define body shape are correlated in the lizard family Gymnophthalmidae and tested for associations between body elongation and climate. We found that variation in the gymnophthalmid body shape involves concomitant changes in different linear morphological traits. For example, trunk elongation is coupled with limb shortening and a reduction in body diameter, and the gradual variation along this axis may be illustrated by less-elongated morphologies exhibiting shorter trunks and longer limbs. Such a morphological gradient confirms the trends previously reported in Gymnophthalmidae [16], [31]. Our analyses also revealed that the variation identified in the gymnophthalmid body shape is associated with climate, with the species from more arid environments being those that are more elongated.

The general patterns of body shape in Gymnophthalmidae coincide with previously identified trends among squamates [9], [16], but variation in body diameter seems peculiar in this group: more-elongated gymnophthalmids are characterized by slimmer bodies, a trend not identified in Anguidae [10] and ambiguously described in Scincidae [2], [36]. It has been argued that variation in body diameter may be constrained by locomotion, as changes in this trait likely influence the effectiveness of the bending forces produced by limbless forms [36]. The evolutionary equation that explains body elongation in Gymnophthalmidae is, however, more complex and probably includes several elements in addition to biomechanics. In this sense, our study incorporates a new element into this equation: the role of climate in body shape variation.

In Gymnophthalmidae, variations in body shape are associated with aridity, a composed index that incorporates the thermal and hydric components of climate. Aridity is associated with high temperatures and low precipitation, likely affecting several additional environmental features, such as the habitat structure, which is defined by the distribution of physical elements along the three-dimensional space in which a given organism lives [37], [38]. This feature may influence the evolution of body shape in gymnophthalmids because divergent environments likely impose distinct demands for organismal performance. Regarding animal locomotion, such contrasting habitats as deserts and rainforests are composed of different substrates, which may impose distinct mechanical demands for running [39]. The structural habitats used by gymnophthalmids differ in the resistance imposed for locomotion [20], and the species distributed in arid environments are those that move on substrates with greater resistance (Figure 3). Furthermore, in Gymnophthalmidae, differences in microhabitat use involve morphological specializations in the head shape because species with more compact heads are associated with microhabitats that offer greater resistance to locomotion [20]. Our data suggests that such differences also result in general changes in body shape. The association between morphology and the structural habitat is well established among squamates [18]–[21], [23], but the incorporation of climate allows the identification of evolutionary relationships on a macro-scale that comprises all of the features influenced by climate.

In addition to the biomechanical elements considered for interpretation of the detected association between body elongation and climate, thermoregulatory components must also be contemplated, as they might also modulate the variation of body shape in Gymnophthalmidae. The preferred body temperatures (Tp) in squamates are positively related with body mass but present a negative relationship with precipitation [40]; thus, larger species in high-rainfall regions tend to have higher Tp values in comparison with small-sized species. Species characterized by small body mass have lower thermal inertia [41], and the variation in shape may contribute an additional component to this relationship because body elongation likely affects the dynamics of the thermal exchange between the organism and its surrounding environment. If the maintenance of thermal preference is under selection in Gymnophthalmidae, then less-elongated bodies may be favored in high-rainfall regions. Forthcoming information about the preferred body temperatures of species from regions with different levels of aridity may allow explicit testing of this hypothesis.

The relationship between body elongation and climate has not been explored previously, thus our study introduces new perspectives for understanding how morphological diversification occurs, with a special focus on the role of different environmental parameters in the evolution of body form. Either directly or indirectly, climate seems to be a key element affecting the evolution of body shape in Gymnophthalmidae, without dismissing the contribution of other biological traits for morphological diversification. Although the role of some of these features may only be evaluated based on the information of behavior and natural history, which are still unavailable for most gymnophthalmids, other explanations for the evolution of body elongation in squamates can already be rejected for Gymnophthalmidae. For example, the elongation-miniaturization hypothesis suggests that the evolution of body elongation results from selective pressures for clutch-size preservation in a miniaturized body [1]; however, despite their small size and regardless of their body shape, clutch size in gymnophthalmids is fixed at two eggs [33], [42], [43]. A similar scenario is observed in pygopodids, which retain the same clutch size despite the evolution of elongated species from a lacertiform ancestral gecko [11]. Assembling additional ecological data will certainly contribute to the understanding of the complexity that underlies the evolution of body elongation in Gymnophthalmidae. Indeed, the present study is pivotal in the establishment of a connection between morphology and a broader natural component, climate. Moreover, the association between body elongation and aridity introduces new questions about the spatial distribution of morphological variation in squamates. For example, we detected that the more-elongated gymnophthalmids are often associated with arid environments, a pattern identified on a large geographical scale that was not accessible from focusing on microhabitat use. Many subsequent questions can be derived from this pattern, with the most general inquiry being the geographical distribution of elongated squamates along climatic gradients.

## Methods

### Morphometric data

The present study employed preserved specimens of 45 gymnophthalmid species (Tables S1 and S2) available at three Brazilian herpetological collections: Museu de Zoologia da Universidade de São Paulo (MZUSP), Coleção Herpetológica da Universidade de Brasília (CHUNB) and personal collection of Dr. MTU Rodrigues (University of São Paulo, specimens not yet deposited at MZUSP). The morphometric data consisted of the following nine measurements obtained using digital calipers (to the nearest 0.01 mm): snout-vent length (SVL - distance from the tip of the snout to posterior end of the cloaca); trunk length (TL - distance from the posterior end of the ventral head scales to the posterior end of the cloaca); anterior and posterior limb lengths (ALL and PLL, respectively - distances from the insertion of the fully extended limbs to the tip of the claw of the longest digit); head length (HL - distance from the tip of the snout to the posterior dorsal head scale, which was generally the interparietal); head width (HW - measurement of the widest portion of the head); head height (HH - measurement of the highest portion of the head); and pelvic girdle width and pelvic girdle height (PGW and PGH respectively - both dimensions measured anteriorly to the insertion of the hindlimbs). Only adults were measured in order to minimize eventual effects of ontogeny; however, both males and females were included due to the limitations in the number of individuals for some species. Even so, we expect that if there is any intraspecific differences regarding sexual dimorphism, it would be irrelevant when compared to the interspecific differences considered, thus having little impact on the results. The average sample size per species was 11.1 individuals; for approximately half species considered we measured 15 to 20 individuals; some rare species, however, were represented by few specimens among the collections used (Table S1), which necessitated combining data from different populations for most of the species studied. The proportion of specimens available that exhibited intact tails was small, and restricting our study to individuals with well-preserved tails would have considerably decreased our sample sizes. Therefore, tail measurements were excluded from the analyses. Our study did not involve the capture or manipulation of live animals, and, therefore, there was no need of approval from the ethics committee because the measurements were obtained from fixed specimens belonging to herpetological collections.

### Climatic Data

The morphometric patterns detected in Gymnophthalmidae were tested for associations with climate, and the occurrence records of the specimens measured were used to extract the climatic data. We chose the climatic variables that better represented central tendency and variation in the thermal and precipitation regimes that gymnophthalmids are exposed to, including climatic extremes. Climatic elements were extracted from Worldclim data layers [44] (available at http://www.worldclim.org, accessed 2012 Oct 18) using DIVA-GIS [45] version 7.1 and treated as single variables in linear models. These variables were Annual Mean Temperature, Mean Diurnal Range, Maximal Temperature of Warmest Month, Minimal Temperature of Coldest Month, Temperature Seasonality, Annual Precipitation, Precipitation of Wettest Quarter, Precipitation of Driest Quarter, and Precipitation Seasonality. Moreover, two additional variables were extracted from the International Water Management Institute (IWMI) World Water and Climate Atlas (available at http://www.iwmi.cgiar.org, accessed 2012 Oct 18), the Highest Monthly Mean Temperature (Tmax) and Lowest Monthly Mean Temperature (Tmin), which were combined with Annual Precipitation (AnnPrec) to calculate a composite variable, named index of aridity (logQ). This index was the same used by Oufiero et al. [25], according to Emberger (cited by [46]). Lower values of logQ correspond to more arid environments and it was calculated according to the following equation: (Q) = AnnPrec/[(Tmax+Tmin)*(Tmax-Tmin)]*1000.

The climatic data associated with those species represented by more than one population were averaged among the locations.

### Statistical Analyses

All of the statistical analyses were conducted using R version 2.14.1 [47] in RStudio (version 0.94.110). Some of these analyses were performed using a phylogenetic framework, which requires the use of a topology representing the relationships among the lineages. We combined two phylogenetic hypotheses available for gymnophthalmids into a single topology: Pellegrino et al. [31], for the overall relationships among the species, and Kohlsdorf & Wagner [48], for the relationships among the *Bachia* species not included in Pellegrino et al. [31]. Some relationships were modified in the topology to accommodate the specificities of our dataset. For example, we measured *Leposoma guianensis*, which was not included in any of the phylogenetic hypotheses available; this species was assumed to be a sister group of *L. percarinatum* (instead of *L. oswaldoi* [49]). Moreover, based on Rodrigues & Santos [50], we considered *Scriptosaura catimbau* as a sister group of *Calyptommatus* because this species was recently described and does not appear in any of the formal phylogenetic hypotheses proposed for the group.

The statistical analyses performed in a phylogenetic framework typically incorporate a topology with branch lengths proportional to the expected variance for the evolution of the analyzed traits (reviewed in [51]). Although some estimates of divergence time or genetic distance are available for specific lineages of Gymnophthalmidae [9], their use would require elimination of a significant number of species from our dataset (e.g. *Anotosaura vanzolinia, Bachia monodactylus, B. panoplia, B. scolecoides, Colobosaura mentalis, Colobosauroides cearensis, Gymnophthalmus vanzoi, Leposoma guianensis, Micrablepharus atticolus, Neusticurus bicarinatus, N. juruazensis, Placosoma cordylinum, Prionodactylus oshaughnessyi, Scriptosaura catimbau*). Given that the use of arbitrary branch lengths is common and well-supported in the literature using Comparative Methods [20]–[22],[25],[51]–[55], we maintained all species measured in our dataset and performed the statistical analyses using arbitrary branch lengths. The adequacy of four different methods for attributing branch lengths was tested following the diagnostics proposed by Garland et al. [52]: 1) All Equal One, 2) Pagel [53], 3) Grafen [54] and 4) Nee (cited in [55]). These diagnostics consist in plotting the absolute value of each standardized independent contrast versus the square root of the sum of its branch lengths, which represent its standard deviation. The method of Grafen appeared to be the most adequate, as indicated by the absence of statistically significant trends in all diagnostic plots produced using these arbitrary branch lengths. The topologies and branch lengths and the diagnostic plots of independent contrasts were built and inspected using Mesquite v2.74 [56] with the PDAP:PDTREE v1.15 [57] module for Mac OSX. The topology adopted was used to phylogenetically size-correct the morphometric variables, following Revell [58], prior to the statistical analyses.

The morphological patterns were investigated considering two main questions: 1) how the variation in linear traits defines the body shape and 2) how the body shape relates to climate. The first question was evaluated using a phylogenetic principal component analysis (PCA; [58]) implemented using the R package phytools [59]. We applied the Kaiser-Guttman criterion and retained the principal components with eigenvalue higher than 1.0 [60]. Our second question investigated how the body shape in Gymnophthalmidae relates to climate, which was tested using phylogenetic linear models. Traditionally, randomization tests of phylogenetic signals are used in such frameworks, but we chose to examine the phylogenetic regression via generalized least squares with the simultaneous estimation of the phylogenetic signal (Pagel's λ, PGLSλ; [35]). The PGLSλ model is similar to a Brownian motion model of evolution but allows for the transformation of branch lengths as it estimates the parameter λ. This parameter (λ) represents the amount of phylogenetic signal in the regression residuals: it ranges from zero to 1.0, with a value closer to zero equivalent to a star phylogeny in which no phylogenetic signal is detectable, and a value closer to 1.0 comparable to a hierarchical phylogeny defined by Brownian motion [61].

The relationship between body elongation and climate was tested using our morphological descriptor resulting from the phylogenetic PCA (morphPC), which was regressed against each of the climatic variables (all single variables extracted from Worldclim and the composite variable logQ) using PGLSλ models. Because body elongation clearly divided the species in two distinct groups (Figure 2), we introduced a new variable: the elongation group (more versus less elongated, EgroupPC). Therefore, we tested a total of 31 models relating body elongation and climate (Table S3) and used AIC as a heuristic indicator of model support based on the likelihood. The models tested followed three main categories: 1) linear models testing for the relationship between morphPC and climatic variables, 2) covariation models relating morphPC with climatic variables and EgroupPC, and 3) the same covariation models with the inclusion of the interaction effects between climatic variables and EgroupPC. We followed Burnham & Anderson [62] and also computed the AICc, which is the AIC corrected for sample size. Smaller AICcs indicate those models with the best fit, and those models within 2 units of the best model are considered to have substantial support [62].

Possible associations between logQ and soil characteristics in which gymnophthalmid species occur were also investigated by regressing a microhabitat index (MI) against logQ. The microhabitat index was calculated by Barros et al. [20] for the same gymnophthalmid species we studied. It considers the proportion of each microhabitat used by a given species together with the force of resistance to displacement that was empirically estimated for each substrate. Thus, MI is proportional to the resistance imposed for locomotion by the substrates gymnophthalmids use. It ranges from 0 to 1, and higher values of MI represent frequent use of microhabitats that offer greatest resistance to displacement. It is important to note that most of the museum specimens measured in the present study were the same used by Barros et al. [20]; even when there were inconsistencies among the populations sampled between the two studies, collection sites for each species were always located in the same Brazilian federative unit.

## Supporting Information

Table S1 Morphological traits (means ± standard errors, all in mm) and scores of morphological principal component for lizards of the family Gymnophthalmidae. (DOCX)

Table S2 List of specimens of Gymnophthalmidae examined. (DOCX)

Table S3 Comparisons of linear models testing the effects of climate variables on morphological component (morphPC) with interaction of elongation groups (EgroupPC). (DOCX)
