# Supplementary material for: Evolution of Body Elongation in Gymnophthalmid Lizards: Relationships with Climate
Source: PLoS One. 2012 Nov 14;7(11):e49772. doi: 10.1371/journal.pone.0049772 (PMC3498171; doi:10.1371/journal.pone.0049772)
Supplement: Table S1 — (DOCX) [file pone.0049772.s001.docx]

Table S1 Morphological traits (means ± standard errors, all in mm) and scores of morphological principal component for lizards of the family Gymnophthalmidae.

| **Species** | **N** | **Snout-vent length** | **Trunk** | **Pelvic girdle height** | **Pelvic girdle width** | **Head length** | **Head height** | **Head width** | **Forelimb length** | **Hindlimb length** | **morphPC** |
| --- | --- | --- | --- | --- | --- | --- | --- | --- | --- | --- | --- |
| Prionodactylus oshaughnessyi | 19 | 44.27 + 0.60 | 35.45 + 0.62 | 5.34 + 0.15 | 4.93 + 0.09 | 10.45 + 0.13 | 4.93 + 0.15 | 6.19 + 0.15 | 12.80 + 0.21 | 17.39 + 0.30 | -1.0522 |
| *Prionodactylus argulus* | 2 | 29.53 + 1.30 | 22.69 + 1.36 | 2.48 + 0.07 | 3.22 + 0.13 | 8.03 + 0.11 | 3.24 + 0.01 | 4.48 + 0.04 | 9.49 + 0.68 | 12.06 + 0.78 | -0.5867 |
| *Cercosaura ocellata* | 16 | 49.82 + 1.13 | 39.92 + 0.94 | 5.98 + 0.13 | 5.55 + 0.17 | 11.25 + 0.17 | 5.25 + 0.10 | 6.67 + 0.13 | 16.55 + 0.44 | 22.95 + 0.62 | -1.2929 |
| *Prionodactylus eigenmanni* | 19 | 40.84 + 0.80 | 31.65 + 0.72 | 4.33 + 0.11 | 4.48 + 0.09 | 10.50 + 0.14 | 4.81 + 0.10 | 6.21 + 0.09 | 14.15 + 0.18 | 19.01 + 0.29 | -1.2809 |
| *Pantodactylus quadrilineatus* | 19 | 36.87 + 0.78 | 29.76 + 0.68 | 3.62 + 0.13 | 3.86 + 0.09 | 8.46 + 0.14 | 3.94 + 0.11 | 5.09 + 0.11 | 10.84 + 0.20 | 14.30 + 0.26 | -0.5474 |
| *Neusticurus juruazensis* | 2 | 50.16 + 1.65 | 40.78 + 2.62 | 6.46 + 0.10 | 5.70 + 0.18 | 10.56 + 0.33 | 5.94 + 0.07 | 7.56 + 0.09 | 16.25 + 0.25 | 24.98 + 0.62 | -1.5514 |
| *Neusticurus bicarinatus* | 18 | 86.03 + 0.00 | 67.94 + 0.00 | 10.84 + 0.00 | 11.01 + 0.00 | 19.77 + 0.00 | 10.57 + 0.00 | 12.25 + 0.00 | 26.94 + 0.00 | 40.53 + 0.00 | -3.4369 |
| *Neusticurus rudis* | 1 | 53.37 | 41.62 | 5.79 | 5.67 | 13.6 | 6.87 | 8.48 | 17.65 | 28.01 | -2.0643 |
| *Placosoma cordylinum* | 16 | 41.56 + 1.58 | 32.16 + 1.42 | 3.26 + 0.16 | 4.29 + 0.19 | 9.97 + 0.31 | 3.77 + 0.14 | 5.62 + 0.20 | 11.65 + 0.40 | 16.61 + 0.62 | -0.5892 |
| *Placosoma glabellum* | 11 | 44.97 + 1.92 | 35.54 + 1.67 | 4.22 + 0.26 | 4.87 + 0.29 | 9.99 + 0.27 | 4.34 + 0.15 | 5.88 + 0.24 | 13.22 + 0.45 | 18.11 + 0.62 | -0.7272 |
| *Bachia dorbignyi* | 16 | 65.25 + 1.63 | 60.12 + 1.60 | 2.55 + 0. 07 | 3.04 + 0.08 | 6.50 + 0.09 | 3.15 + 0.06 | 4.10 + 0.08 | 2.39 + 0.04 | 1.02 + 0.03 | 3.5693 |
| *Bachia flavescens* | 1 | 68.98 | 61.87 | 3.47 | 3.98 | 7.88 | 4.27 | 5.38 | 3.85 | 4.03 | 2.8528 |
| *Bachia monodactylus* | 9 | 61.32 + 1.26 | 56.47 + 1.27 | 2.99 + 0.15 | 3.40 + 0.08 | 6.58 + 0.14 | 3.30 + 0.06 | 3.98 + 0.11 | 2.67 + 0.12 | 2.41 + 0.21 | 3.0654 |
| *Bachia panoplia* | 14 | 68.89 + 1.91 | 61.85 + 1.84 | 3.74 + 0.14 | 5.38 + 0.10 | 8.03 + 0.15 | 4.19 + 0.1 | 5.35 + 0.11 | 3.38 + 0.07 | 4.58 + 0.26 | 2.6337 |
| *Bachia bresslaui* | 6 | 83.79 + 3.97 | 75.91 + 3.54 | 4.62 + 0.22 | 4.97 + 0.24 | 8.79 + 0.29 | 4.31 + 0.26 | 5.22 + 0.23 | 2.61 + 0.21 | 3.01 + 0.27 | 3.8610 |
| *Bachia scolecoides* | 2 | 66.20 + 7.22 | 60.56 + 5.60 | 3.33 + 0.64 | 3.68 + 0.35 | 7.27 + 0.40 | 3.62 + 0.43 | 4.74 + 0.55 | 2.96 + 0.23 | 3.81 + 0.18 | 3.0791 |
| *Colobosauroides cearensis* | 1 | 36.71 | 30.07 | 2.80 + 0.80 | 2.99 + 0.80 | 6.46 | 3.15 | 4.29 | 6.31 | 9.92 | 0.4203 |
| *Anotosaura vanzolinia* | 20 | 39.67 + 0.62 | 34.64 + 0.60 | 2.59 + 0.06 | 2.94 + 0.05 | 5.82 + 0.07 | 2.85 + 0.05 | 3.80 + 0.03 | 4.13 + 0.09 | 8.49 + 0.09 | 1.1494 |
| *Leposoma guianensis* | 7 | 34.79 + 0.56 | 29.70 + 0.49 | 4.01 + 0.11 | 4.05 + 0.08 | 7.48 + 0.14 | 3.97 + 0.13 | 5.17 + 0.09 | 8.65 + 0.19 | 13.53 + 0.22 | -0.4877 |
| *Leposoma percarinatum* | 16 | 32.51 + 0.53 | 26.64 + 0.42 | 3.69 + 0. 80 | 3.85 + 0.07 | 6.85 + 0.07 | 3.43 + 0.07 | 4.46 + 0.06 | 9.04 + 0.17 | 13.33 + 0.18 | -0.4342 |
| *Arthrosaura kockii* | 7 | 39.82 + 2.53 | 31.63 + 2.27 | 4.30 + 0.36 | 5.42 + 0.39 | 9.09 + 0.44 | 4.61 + 0.30 | 6.60 + 0.36 | 13.51 + 0.86 | 20.46 + 1.33 | -1.3399 |
| *Arthrosaura reticulata* | 20 | 51.10 + 1.24 | 41.13 + 1.01 | 5.59 + 0.21 | 6.21 + 0.16 | 10.46 + 0.23 | 5.46 + 0.20 | 7.48 + 0.23 | 12.95 + 0.35 | 20.62 + 0.47 | -1.1199 |
| *Micrablepharus maximiliani* | 20 | 35.35 + 0.41 | 30.26 + 0.39 | 3.47 + 0.07 | 4.24 + 0.05 | 6.57 + 0.07 | 3.72 + 0.03 | 4.82 + 0.04 | 9.55 + 0.11 | 14.04 + 0.17 | -0.2598 |
| *Micrablepharus atticolus* | 12 | 36.34 + 1.05 | 31.68 + 0.98 | 3.58 + 0.14 | 4.78 + 0.11 | 6.81 + 0.13 | 3.54 + 0.07 | 4.93 + 0.06 | 9.65 + 0.16 | 14.37 + 0.22 | -0.2415 |
| *Tretioscincus agilis* | 11 | 38.59 + 1.07 | 33.01 + 1.03 | 3.87 + 0.13 | 4.98 + 0.13 | 7.31 + 0.11 | 3.76 + 0.09 | 5.35 + 0.10 | 10.85 + 0.25 | 16.51 + 0. 27 | -0.4271 |
| *Procellosaurinus erythrocercus* | 6 | 27.45 + 0.73 | 23.01 + 0.75 | 2.22 + 0.06 | 3.06 + 0.07 | 5.21 + 0.14 | 2.52 + 0.10 | 3.62 + 0.12 | 6.27 + 0.19 | 10.52 + 0.20 | 0.1279 |
| *Procellosaurinus tetradactylus* | 4 | 27.54 + 0.96 | 23.73 + 0.83 | 2.16 + 0.06 | 3.12 + 0.15 | 4.60 + 0.33 | 2.43 + 0.16 | 3.47 + 0.19 | 6.38 + 0.13 | 11.41 + 0.41 | 0.2456 |
| *Vanzosaura rubricauda* | 20 | 31.68 + 0.56 | 27.14 + 0.51 | 2.77 + 0.09 | 3.63 + 0.10 | 5.89 + 0.10 | 2.96 + 0.06 | 3.98 + 0.05 | 8.54 + 0.16 | 13.28 + 0.28 | 0.0025 |
| *Nothobachia ablephara* | 9 | 48.28 + 1.67 | 44.04 + 1.52 | 2.61 + 0.12 | 3.05 + 0.12 | 5.31 + 0.19 | 2.66 + 0.09 | 3.24 + 0.11 | 1.61 + 0.11 | 5.56 + 0.25 | 2.3094 |
| *Scriptosaura catimbau* | 19 | 48.17 + 0.78 | 43.41 + 0.73 | 2.78 + 0.08 | 3.33 + 0.07 | 5.82 + 0.08 | 2.76 + 0.05 | 3.28 + 0.06 | 0.00 + 0.00 | 2.32 + 0.07 | 2.2816 |
| *Calyptommatus leiolepis* | 20 | 59.95 + 1.11 | 54.82 + 1.05 | 3.89 + 0.10 | 4.27 + 0.10 | 6.52 + 0.13 | 3.52 + 0.05 | 4.33 + 0.07 | 0.00 + 0.00 | 3.88 + 0.06 | 2.6457 |
| *Calyptommatus nicterus* | 20 | 59.51 + 0.87 | 54.40 + 0.83 | 3.30 + 0.07 | 3.95 + 0.05 | 5.97 + 0.07 | 3.14 + 0.03 | 4.13 + 0.04 | 0.00 + 0.00 | 3.96 + 0.05 | 2.8751 |
| *Calyptommatus sinebrachiatus* | 20 | 56.34 + 0.86 | 51.61 + 0.81 | 3.25 + 0.10 | 3.67 + 0.08 | 5.92 + 0.07 | 3.09 + 0.04 | 3.88 + 0.05 | 0.00 + 0.00 | 3.35 + 0.06 | 2.7265 |
| *Gymnophthalmus leucomystax* | 15 | 34.30 + 0.99 | 30.35 + 0.89 | 2.73 + 0.08 | 3.64 + 0.10 | 5.75 + 0.12 | 2.76 + 0.06 | 4.05 + 0.07 | 8.72 + 0.11 | 11.31 + 0.19 | 0.3771 |
| *Gymnophthalmus vanzoi* | 2 | 35.35 + 2.54 | 31.36 + 2.39 | 2.25 + 0.04 | 3.60 + 0.03 | 6.06 + 0.02 | 2.92 + 0.27 | 4.17 + 0.08 | 7.78 + 0.63 | 11.49 + 0.08 | 0.4939 |
| *Psilophthalmus paeminosus* | 10 | 28.13 + 1.23 | 23.42 + 0.87 | 2.07 + 0.13 | 2.73 + 0.07 | 4.68 + 0.09 | 2.31 + 0.11 | 3.20 + 0.09 | 4.67 + 0.11 | 8.81 + 0.21 | 0.4823 |
| *Colobosaura modesta* | 20 | 43.70 + 1.33 | 36.65 + 1.13 | 3.75 + 0.16 | 4.84 + 0.17 | 8.99 + 0.22 | 4.41 + 0.18 | 6.04 + 0.24 | 10.45 + 0.28 | 17.74 + 0.50 | -0.4338 |
| *Acratosaura mentalis* | 3 | 53.63 + 4.66 | 45.82 + 4.26 | 4.10 + 0.42 | 5.46 + 0.41 | 10.14 + 0.54 | 4.68 + 0.45 | 6.57 + 0.48 | 12.33 + 0.80 | 20.90 + 1.44 | -0.1109 |
| *Iphisa elegans* | 7 | 44.26 + 2.40 | 38.04 + 2.17 | 3.75 + 0.28 | 4.55 + 0.29 | 8.55 + 0.42 | 4.27 + 0.18 | 5.92 + 0.32 | 10.28 + 0.56 | 16.53 + 1.71 | -0.1565 |
| *Heterodactylus imbricatus* | 8 | 98.28 + 2.96 | 87.69 + 2.49 | 8.00 + 0.38 | 8.53 + 0.41 | 10.07 + 0.39 | 8.59 + 0.46 | 10.66 + 0.63 | 13.83 + 0.62 | 25.15 + 0.87 | 1.3021 |
| *Colobodactylus taunay* | 7 | 47.94 + 2.86 | 41.54 + 2.54 | 3.72 + 0.28 | 4.73 + 0.22 | 8.03 + 0.33 | 4.39 + 0.20 | 5.95 + 0.29 | 9.99 + 0.52 | 17.69 + 1.08 | 0.1355 |
| *Colobodactylus dalcyanus* | 1 | 38.48 | 31.90 | 3.18 | 4.31 | 7.09 | 3.90 | 4.99 | 8.51 | 12.89 | -0.0991 |
| *Rachisaurus brachylepis* | 4 | 52.79 + 4.51 | 45.61 + 4.47 | 2.92 + 0.17 | 3.73 + 0.30 | 7.78 + 0.52 | 3.41+ 0.22 | 4.82 + 0.37 | 7.70 + 0.31 | 11.90 + 0.92 | 1.3705 |
| *Alopoglossus carinicaudatus* | 9 | 54.80 + 1.21 | 46.49 + 1.06 | 6.64 + 0.27 | 7.37 + 0.17 | 10.96 + 0.22 | 6.50 + 0.18 | 8.38 + 0.15 | 7.76 + 0.16 | 19.04 + 0.18 | -1.0901 |
| *Alopoglossus artriventris* | 17 | 43.13 + 0.72 | 33.91 + 0.62 | 5.22 + 0.10 | 5.70 + 0.11 | 9.94 + 0.15 | 5.30 + 0.12 | 7.01 + 0.14 | 13.55 + 0.23 | 20.72 + 0.34 | -1.5461 |

N = number of individuals. Elongation index is given by Snout-vent length divided by Pelvic girdle width. morphPC = morphological principal component.
