# Supplementary material for: Evolution of Body Elongation in Gymnophthalmid Lizards: Relationships with Climate
Source: PLoS One. 2012 Nov 14;7(11):e49772. doi: 10.1371/journal.pone.0049772 (PMC3498171; doi:10.1371/journal.pone.0049772)
Supplement: Table S2 — (DOCX) [file pone.0049772.s002.docx]

Table S2 List of specimens of Gymnophthalmidae examined. Species are listed alphabetically. Museum acronyms are as follows: Museu de Zoologia da Universidade de São Paulo, MZUSP; Coleção Herpetológica da Universidade de Brasília, CHUNB; Dr. MTU Rodrigues personal collection, MTR.

| **Species** | **Voucher** |
| --- | --- |
| *Aloploglossus artriventris* | MZUSP 59035, 28291, 13916, 13917, 13919-13921, 46789, 13061, 28339, 28318, 56648, 56649, 56633-56635, 56661, 39944 |
| *Aloploglossus carinicaudatus* | MZUSP 57637, 67052, 53792, 53791, 53794, 89344, 89342, 50630, 66144 |
| *Anotosaura vanzolinia* | MZUSP 77829-77832, 77834, 95344, 95345, 95348, 95316, 78151, 78178, 78180, 95343, 95349, 95315-95318, 95320-95322 |
| *Arthrosaura kockii* | MZUSP 13965, 90065, 78181, 98614, 98380-98382 |
| *Arthrosaura reticulata* | MZUSP 41775, 53815, 54367, 67582, 30791, 57621, 52502, 52497-52499, 89355, 89358, 89359, 89360-89362, 89354, 82644, 82646, 43039 |
| *Bachia bresslaui* | CHUNB 25851, 50258, 51322, 39116, 11073, 11075 |
| *Bachia dorbigny* | MZUSP 97920, 97915, 97912, 82421, 82422, 82647, 81604, 2063, 82423, 97917, 97914, 97918, 97916, 97919, 97921, 97913 |
| *Bachia flavescens* | MZUSP 68930 |
| *Bachia monodactyla* | MZUSP 2843, 8353, 10912, 26007, 471, 8352, 81686, 49350, 51642 |
| *Bachia panoplia* | MZUSP 43008, 57562, 57561, 58814, 60598, 57852, 49285, 57638, 26008, 60597, 57639, 10910, 55706, 55707 |
| *Bachia scolecoides* | MZUSP 38374; CHUNB 47062 |
| *Calyptommatus leiolepis* | MZUSP 75846, 75847, 75648, 75858, 75774, 75877, 75884, 75651, 75820, 75767, 75669, 75930, 75650, 75659, 93371, 93387, 93373 |
| *Calyptommatus nicterus* | MZUSP 93370, 93375, 75729, 70485, 70660, 70642, 70908, 70627, 70601, 70546, 70971, 70832, 70661, 70603, 70966, 70681, 70718, 70707, 71002, 70489, 70871, 70646, 70889 |
| *Calyptommatus sinebrachiatus* | MZUSP 76764, 76751, 76771, 76762, 76743, 76745, 76738, 76739, 76774, 76767, 76759, 76763, 76753, 76757, 76749, 76758 |
| *Cercosaura ocellata* | MZUSP 62843, 41776, 66394, 21223, 67586, 73457, 73458, 72667, 91697, 91698, 91701, 91703, 91692, 91693, 91694, 91695 |
| *Colobodactylus dalcyanus* | MZUSP 78417 |
| *Colobodactylus taunay* | MZUSP 4705, 89798, 90010, 91421, 88173, 91684, 91470 |
| *Acratosaura mentalis* | MZUSP 76218, 76219, 74224 |
| *Colobosaura modesta* | MZUSP 80067, 80063, 80066, 80052, 80080, 80043, 80556, 80553, 80555, 80837, 80841, 80078, 80073, 80840, 88702, 88707, 88690, 88692, 88705, 89592 |
| *Colobosauroides cearensis* | MZUSP 79214 |
| *Gymnophtalmus leucomystax* | MZUSP 72705, 72703, 72701, 72710, 72714, 72706, 72692, 72700, 72696, 72689, 73065, 73061, 73066, 70361, 72704 |
| *Gymnophtalmus vanzoi* | MZUSP 95226, 95225 |
| *Heterodactylus imbricatus* | MZUSP 94047, 94048, 94050, 89185, 88871, 57971, 89186, 88147 |
| *Iphisa elegans* | MZUSP 82429, 82428, 8354, 88464, 56713, 91383, 91385 |
| *Leposoma guianensis* | MZUSP 53800, 53804, 53807, 53857, 36017, 53712, 36015 |
| *Leposoma percarinatum* | MZUSP 79391, 77465, 64611, 53686, 66399, 52520, 57622, 66405, 66972, 66971, 66705, 66350, 29382, 94863, 94864, 94865 |
| *Micrablepharus atticolus* | MZUSP 95255, 95264, 95257, 95251, 95256, 95258, 95266, 95252, 95253, 95254, 91886, 81201 |
| *Micrablepharus maximiliani* | MZUSP 80870, 80863, 80904, 80865, 80853, 80903, 80876, 80915, 80864, 80891, 80866, 80892, 80860, 80877, 80902, 80907, 80899, 80889, 80887, 80871 |
| *Neusticurus bicarinatus* | MZUSP 53644, 79403, 88443, 8073, 6310, 6311, 6312, 6313, 55596, 81738, 81739, 82474, 81659, 81660, 95172, 630, 9006, 89966; |
| *Neusticurus juruazensis* | MZUSP 88653, 88654 |
| *Neusticurus rudis* | MZUSP 78141 |
| *Notobachia ablephara* | MZUSP 76874, 76871, 76872, 76873, 76876, 76901, 76906, 76004, 76886 |
| *Pantodactylus quadrilineatus* | MZUSP 4767-4769, 54840, 4760, 29604, 4755, 4759, 40727, 40728, 11881, 55592, 13988, 4787 , 2280, 6307, 4750, 4754, 3187 |
| *Placosoma cordylinum* | MZUSP 78219, 8805, 74906, 17004, 78421, 78422, 78423, 89960, 89961, 89962, 8806, 3477, 78425, 3480, 3485, 3489; |
| *Placosoma glabellum* | MZUSP 95126, 792, 95434, 78220, 89980, 681, 62392, 74907, 89137, 89138, 76281 |
| *Prionodactylus argulus* | MZUSP 55687, 55688 |
| *Prionodactylus eigenmanni* | MZUSP 61939-61943, 66342-66345, 81690, 81691, 82739, 82741, 82742, 82744, 82746, 81587, 81588, 82856 |
| *Prionodactylus oshaughnessyi* | MZUSP 47467-47472, 47475, 47479, 47482, 47497, 47510, 47514, 47515, 47517, 41779, 10903, 53541, 53545 |
| *Procellosaurinus erythrocercus* | MZUSP 75317, 77876, 74938, 74934, 76263, 95159 |
| *Procellosaurinus tetradactylus* | MZUSP 76409, 91914, 91909, 91910 |
| *Psilophtalmus paeminosus* | MZUSP 74960, 74963, 74957, 76410, 79758, 77985, 77983, 74961, 74952, 74954 |
| *Rhachisaurus brachylepis* | MZUSP 40089, 72375, 72376, 72377 |
| *Scriptosaura catimbau* | MZUSP 98059-98063, 98067, 98069, 98070, 98072, 98074, 98077; MTR 14101, 14102, 14107, 14110, 14112, 14114, 14115, 14116 |
| *Tretioscincus agilis* | MZUSP 12503, 12426, 12560, 12438, 12444, 35256, 35278, 31399, 35240, 12394, 35313 |
| *Vanzosaura rubricauda* | MZUSP 94123-94126, 94129, 94131, 94132, 94134-94136, 94138-94140, 94142, 76960, 76933, 76971, 76973, 76939, 76975 |
