# Supplementary material for: Evolution of Body Elongation in Gymnophthalmid Lizards: Relationships with Climate
Source: PLoS One. 2012 Nov 14;7(11):e49772. doi: 10.1371/journal.pone.0049772 (PMC3498171; doi:10.1371/journal.pone.0049772)
Supplement: Table S3 — (DOCX) [file pone.0049772.s003.docx]

Table S3. Comparisons of linear models testing the effects of climate variables on morphological component (morphPC) with interaction of elongation groups (EgroupPC). Significant values (P<0.05) indicated by an asterisk (*).

| **Model** | **Parameter** | **Slope** | **P** | **lambda** | **AICc** | **ΔAICc** |
| --- | --- | --- | --- | --- | --- | --- |
| morphPC ~ logQ x EgroupPC | log Q | -1.451743 | <0.00001* | 0.9450776 | 93.5 | 0 |
|  | EgroupPC | -1.298553 | 0.6563 |  |  |  |
|  | log.Q x EgroupPC | 1.056726 | 0.1975 |  |  |  |
| morphPC ~ logQ + EgroupPC | log Q | -1.282886 | <0.00001* | 0.9610938 | 93.8 | 0.3 |
|  | EgroupPC | 2.429285 | <0.00001* |  |  |  |
| morphPC ~ EgroupPC |  | -0.3659299 | 0.7211 | 0.9882093 | 107.3 | 13.8 |
| morphPC ~ Annual Mean Temperature + EgroupPC | Annual Mean Temperature | -0.1155913 | 0.0064* | 0.947564 | 107.7 | 14.2 |
|  | EgroupPC | 2.7546848 | <0.00001* |  |  |  |
| morphPC ~ logQ |  | -1.245647 | 0.0003* | 1.002787 | 107.8 | 14.3 |
| morphPC ~ Temperature Seasonality + EgroupPC | Temperature Seasonality | 0.0064392 | 0.0004* | 0.9747767 | 107.9 | 14.4 |
|  | EgroupPC | 2.6250464 | <0.00001* |  |  |  |
| morphPC ~ Min Temperature of Coldest Month + EgroupPC | Min Temperature of Coldest Month | -0.0720827 | 0.0104* | 0.9393836 | 109.6 | 16.1 |
|  | EgroupPC | 2.6506762 | <0.00001* |  |  |  |
| morphPC ~ Max Temperature of Warmest Month + EgroupPC | Max Temperature of Warmest Month | -0.0984012 | 0.0234* | 0.9582996 | 109.7 | 16.2 |
|  | EgroupPC | 2.8365144 | <0.00001* |  |  |  |
| morphPC ~ Annual Mean Temperature x EgroupPC | Annual Mean Temperature | -0.105418 | 0.0173* | 0.9502462 | 111.9 | 18.4 |
|  | EgroupPC | 5.336823 | 0.137 |  |  |  |
|  | Annual Mean Temperature x EgroupPC | -0.101315 | 0.4616 |  |  |  |
| morphPC ~ Mean Monthly Temperature Range + EgroupPC | Mean Monthly Temperature Range | -0.0233333 | 0.7449 | 0.9907291 | 113.2 | 19.7 |
|  | EgroupPC | 2.7096077 | 0.0003* |  |  |  |
| morphPC ~ Annual Precipitation + EgroupPC | Annual Precipitation | -0.0006512 | 0.0005* | 0.9597497 | 113.5 | 20 |
|  | EgroupPC | 2.3797037 | <0.0000*1 |  |  |  |
| morphPC ~ Max Temperature of Warmest Month x EgroupPC | Max Temperature of Warmest Month | -0.091824 | 0.0452* | 0.9599894 | 114.3 | 20.8 |
|  | EgroupPC | 4.769097 | 0.2988 |  |  |  |
|  | Max Temperature of Warmest Month x EgroupPC | -0.057613 | 0.6692 |  |  |  |
| morphPC ~ Precipitation of Wettest Month + EgroupPC | Precipitation of Wettest Month | -0.0033232 | 0.0099* | 0.9836571 | 114.5 | 21 |
|  | EgroupPC | 2.3761645 | 0.0003* |  |  |  |
| morphPC ~ Min Temperature of Coldest Month x EgroupPC | Min Temperature of Coldest Month | -0.0686738 | 0.0284* | 0.9410147 | 115.7 | 22.3 |
|  | EgroupPC | 2.9267186 | 0.027* |  |  |  |
|  | Min Temperature of Coldest Month x EgroupPC | -0.0164303 | 0.8131 |  |  |  |
| morphPC ~ Precipitation Seasonality + EgroupPC | Precipitation Seasonality | 0.0124293 | 0.0644 | 0.9302509 | 116 | 22.5 |
|  | EgroupPC | 2.6285539 | <0.00001* |  |  |  |
| morphPC ~ Precipitation of Driest Month + EgroupPC | Precipitation of Driest Month | -0.0069617 | 0.0591 | 0.9590509 | 116.2 | 22.7 |
|  | EgroupPC | 2.5819274 | <0.0001* |  |  |  |
| morphPC ~ Mean Monthly Temperature Range x EgroupPC | Mean Monthly Temperature Range | -0.0579372 | 0.537 | 0.9928087 | 117.6 | 24.1 |
|  | EgroupPC | 1.7849439 | 0.3501 |  |  |  |
|  | Mean Monthly Temperature Range x EgroupPC | 0.0755321 | 0.6005 |  |  |  |
| morphPC ~ 1 |  | -0.00136636 | 0.9992 | 1.006838 | 118.3 | 24.8 |
| morphPC ~ Temperature Seasonality x EgroupPC | Temperature Seasonality | 0.0062279 | 0.0012* | 0.9751126 | 119 | 25.5 |
|  | EgroupPC | 2.4260239 | 0.0028* |  |  |  |
|  | Temperature Seasonality x EgroupPC | 0.002009 | 0.7182 |  |  |  |
| morphPC ~ Temperature Seasonality |  | 0.006561 | 0.0027* | 1.005556 | 122 | 28.5 |
| morphPC ~ Annual Mean Temperature |  | -0.0820844 | 0.087 | 1.002818 | 122.2 | 28.7 |
| morphPC ~ Max Temperature of Warmest Month |  | -0.0582821 | 0.1891 | 1.005314 | 123.4 | 29.9 |
| morphPC ~ Min Temperature of Coldest Month |  | -0.0530518 | 0.1039 | 1.000442 | 123.5 | 30 |
| morphPC ~ Mean Monthly Temperature Range |  | -0.0257356 | 0.733 | 1.007931 | 124 | 30.5 |
| morphPC ~ Precipitation of Wettest Month x EgroupPC | Precipitation of Wettest Month | -0.0037268 | 0.0055* | 0.968875 | 124.2 | 30.7 |
|  | EgroupPC | 1.1136935 | 0.3343 |  |  |  |
|  | Precipitation of Wettest Month x EgroupPC | 0.0075294 | 0.2064 |  |  |  |
| morphPC ~ Precipitation Seasonality x EgroupPC | Precipitation Seasonality | 0.019766 | 0.008* | 0.7582815 | 124.3 | 30.9 |
|  | EgroupPC | 4.561102 | 0.0019* |  |  |  |
|  | Precipitation Seasonality x EgroupPC | -0.021532 | 0.1768 |  |  |  |
| morphPC ~ Precipitation of Wettest Month |  | -0.0036426 | 0.0063* | 1.00467 | 124.6 | 31.1 |
| morphPC ~ Precipitation of Driest Month x EgroupPC | Precipitation of Driest Month | -0.0072137 | 0.0737 | 0.9572372 | 126.3 | 32.8 |
|  | EgroupPC | 2.568426 | 0.0001* |  |  |  |
|  | Precipitation of Driest Month x EgroupPC | 0.0012609 | 0.8983 |  |  |  |
| morphPC ~ Annual Precipitation x EgroupPC | Annual Precipitation | -0.0007351 | 0.0002* | 0.9130247 | 127.8 | 34.3 |
|  | EgroupPC | 1.6065379 | 0.0341* |  |  |  |
|  | Annual Precipitation x EgroupPC | 0.0008301 | 0.1647 |  |  |  |
| morphPC ~ Precipitation of Driest Month |  | -0.00535317 | 0.1641 | 1.001556 | 128.4 | 34.9 |
| morphPC ~ Precipitation Seasonality |  | 0.00447578 | 0.5177 | 1.002736 | 128.7 | 35.2 |
| morphPC ~ Annual Precipitation |  | -0.0006608 | 0.0014* | 1 | 182.6 | 89.1 |
|  |  |  |  |  |  |  |

morphPC = morphological component. EgroupPC = elongation group based on morphPC.
